# Supplementary material for: Data-driven simulator of multi-animal behavior with unknown dynamics via reinforcement learning
Source: iScience. 2026 Jun 1;29(6):116192. doi: 10.1016/j.isci.2026.116192 (PMC13233570; doi:10.1016/j.isci.2026.116192)
Supplement: Document S1. Figures S1 and S2, Tables S1 and S2, and Data S1 [file mmc1.pdf]

iScience, Volume *29*

Supplemental information

Data-driven simulator of multi-animal behavior

with unknown dynamics via reinforcement learning

Keisuke Fujii, Kazushi Tsutsui, Yu Teshima, Makoto Itoh, Naoya Takeishi, Nozomi Nishiumi, Ryoya Tanaka, Shunsuke Shigaki, and Yoshinobu Kawahara

Supplementary Materials

Data S1. Complementary counterfactual prediction results under alternative pretraining settings.

For artificial agents trained without pretraining, path length distributions under Condition 1 (independent reward) and Condition 2 (shared reward) had no significant difference, and counterfactual queries ($1 \to 2$ and $2 \to 1$) did not produce significant shifts in path length (bootstrap 95% CIs overlapped zero; Fig. S1a). For silkmoths trained with pretraining, path length distributions under full versus reduced sensory input similarly had no significant difference, and counterfactual flips obtained no significant shifts (bootstrap 95% CIs overlapped zero; Fig. S1b).

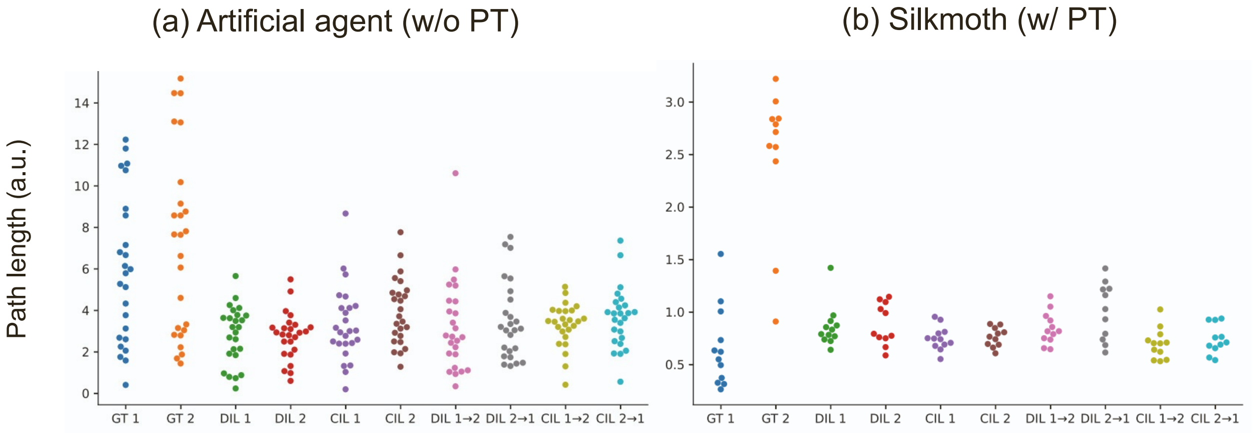

Figure S1: **Counterfactual trajectory examples for (a) artificial agents and (b) silkmoth.** *The layouts for each subfigure are the same as Fig. 5.*

Supplementary Figure

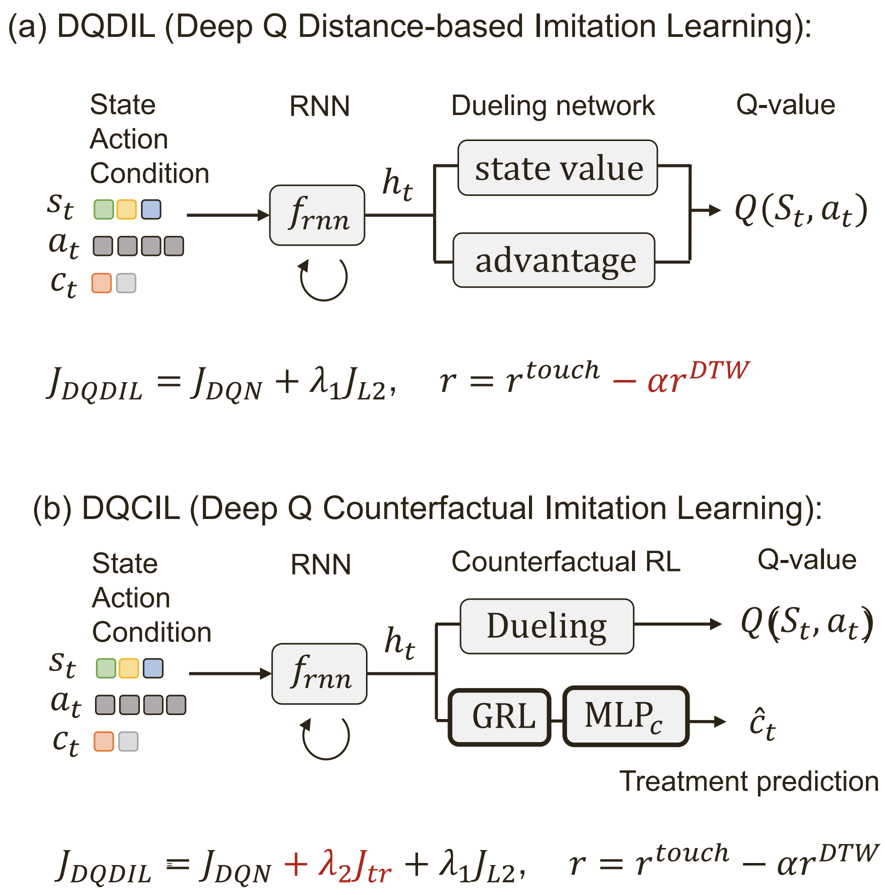

Figure S2: **Overview of DQDIL and DQCIL architectures and objectives.** *(a) DQDIL uses a recurrent encoder f_{rnn} and a dueling Q-network (state-value and advantage heads). The red term highlights the distance-based pseudo-reward component, $-\alpha r^{\text{DTW}}$, which is the key addition in DQDIL. (b) DQCIL extends DQDIL by adding an adversarial condition-prediction branch that consists of a gradient reversal layer (GRL) and a condition classifier MLP_c. The red term $+\lambda_2 J_{\text{tr}}$ denotes the additional objective that differentiates DQCIL from DQDIL, which is used to reduce condition-specific sampling bias and support cue-swapping (counterfactual) queries at inference.*

Supplementary Tables

Table S1: ***Dataset summary and data splits.*** *Episode duration statistics and the data splits (train/validation/test) used for locomotion parameter estimation, offline policy learning, and online policy adjustment.*

Dataset	Episodes	Duration (min/max/total, s)	Locomotion split	Offline split	Online split
Agents	500	0.2 / 17.9 / 2983.8	400/50/50	400/50/50	50/10
Flies	107	0.4 / 47.8 / 425.6	94/13	84/13/10	50/10
Newts	280	2.5 / 50 / 12141.6	240/40	240/20/20	50/10
Silkmoth	60	9.0 / 300 / 6611.5	36/24	36/12/12	36/24

Table S2: ***Training hyperparameters summary.*** *Unified hyperparameters used in offline training and online policy adjustment across domains in DQDIL/DQCIL/DQAAS.*

Hyperparameter	Artificial agents	Flies	Newts	Silkmoth
Offline training				
Epochs	30	30	30	30
λ_1 (L2 regularization)	10^{-5}	10^{-5}	10^{-5}	10^{-5}
Learning rate (offline RL)	10^{-6}	10^{-4}	10^{-4}	10^{-5}
α (DTW pseudo-reward)	10	0.5	0.5	10
λ_2 (counterfactual loss; DQCIL only)	10	N/A	N/A	10
λ_3 (AAS regularizer; DQAAS only)	50	10	10	50
Learning rate (AAS/BC baselines)	10^{-3}	10^{-3}	10^{-3}	10^{-3}
Online policy adjustment				
Training horizon (environment steps)	1,005,000	1,005,000	1,005,000	1,005,000
λ_1 (L2 regularization)	10^{-5}	10^{-5}	10^{-5}	10^{-5}
Learning rate (no pretrain)	10^{-6}	10^{-4}	10^{-4}	10^{-5}
Learning rate (with pretrain)	10^{-6}	10^{-5}	10^{-5}	10^{-7}
Decay duration (steps)	5×10^4	5×10^4	5×10^4	5×10^4
α (with pretrain)	1	0.5	1	1
λ_2 (with pretrain; DQCIL only)	1	N/A	N/A	1
λ_3 (with pretrain; DQAAS only)	10	5	0.1	10
